# Genetic and technological diversity of *Streptococcus thermophilus* isolated from the Saint-Nectaire PDO cheese-producing area

**DOI:** 10.3389/fmicb.2023.1245510

**Published:** 2023-11-14

**Authors:** Anna Grizon, Sebastien Theil, Cecile Callon, Pauline Gerber, Sandra Helinck, Eric Dugat-Bony, Pascal Bonnarme, Christophe Chassard

**Affiliations:** ^1^UMR545 Fromage, INRAE, VetAgro Sup, Université Clermont Auvergne, Aurillac, France; ^2^Pôle Fromager AOP Massif Central, Aurillac, France; ^3^Université Paris-Saclay, INRAE, AgroParisTech, UMR SayFood, Palaiseau, France

**Keywords:** *Streptococcus thermophilus*, diversity, genome comparison, functional properties, autochthonous starter, cheese

## Abstract

*Streptococcus thermophilus* is of major importance for cheese manufacturing to ensure rapid acidification; however, studies indicate that intensive use of commercial strains leads to the loss of typical characteristics of the products. To strengthen the link between the product and its geographical area and improve the sensory qualities of cheeses, cheese-producing protected designations of origin (PDO) are increasingly interested in the development of specific autochthonous starter cultures. The present study is therefore investigating the genetic and functional diversity of *S. thermophilus* strains isolated from a local cheese-producing PDO area. Putative *S. thermophilus* isolates were isolated and identified from milk collected in the Saint-Nectaire cheese-producing PDO area and from commercial starters. Whole genomes of isolates were sequenced, and a comparative analysis based on their pan-genome was carried out. Important functional properties were studied, including acidifying and proteolytic activities. Twenty-two isolates representative of the diversity of the geographical area and four commercial strains were selected for comparison. The resulting phylogenetic trees do not correspond to the geographical distribution of isolates. The clustering based on the pan-genome analysis indicates that isolates are divided into five distinct groups. A Kyoto Encyclopedia of Genes and Genomes (KEGG) functional annotation of the accessory genes indicates that the accessory gene contents of isolates are involved in different functional categories. High variability in acidifying activities and less diversity in proteolytic activities were also observed. These results indicate that high genetic and functional variabilities of the species *S. thermophilus* may arise from a small (1,800 km^2^) geographical area and may be exploited to meet demand for use as autochthonous starters.

## 1. Introduction

*Streptococcus thermophilus* is a thermophilic lactic acid bacteria (LAB) with major economic importance for the dairy industry. Due to its ability to rapidly acidify the milk, it is extensively used for the manufacture of several important fermented dairy products and is considered the second most important species among LAB after *Lactococcus lactis* (Harnett et al., 2022). *Streptococcus thermophilus* is a clonal species that has only recently emerged from a commensal ancestor of the *salivarius* group (Delorme, 2008). This species is part of the genus *Streptococcus* and is the only one that obtained the Generally Recognized as Safe (GRAS) status and the Qualified Presumption of Safety (QPS) status within this genus. Comparative genomic studies on *Streptococcus thermophilus* genomes highlight that this species has lost many virulence-related functions common among pathogenic streptococci. These studies suggest that the *Streptococcus thermophilus* genome has followed a regressive evolution process toward a specialized bacterium dedicated to growth in milk. Comparative genomics also revealed that the dairy *Streptococcus* genome may have been shaped mainly through loss-of-function events and horizontal gene transfer (Bolotin et al., 2004; Hols et al., 2005; Goh et al., 2011).

In the dairy industry, *Streptococcus thermophilus* is used as a starter culture for its capability to rapidly acidify the milk, which is a technological characteristic of major importance to guarantee a good outcome of the dairy process and to enhance food safety by preventing the development of pathogenic bacteria (Rakhmanova et al., 2018). Proteolysis is considered one of the most important enzymatic pathways in the manufacture of many fermented dairy products. In cheese production, proteolysis plays a crucial role in the flavor and texture of cheese by releasing peptides and free amino acids that could undergo secondary reactions (Fox, 1989; Smit et al., 2005).

Over the past few years, changes in the way of life of consumers have imposed requirements for uniformity in the quality of traditional dairy products with high typicality (Settanni and Moschetti, 2014). Due to this growing interest in preserving the typical sensorial properties of traditional cheeses, there is an increasing demand for autochthonous (also called local, indigenous, or wild) and specific strains to replace the well-defined industrial starter strains largely widespread and extensively used in dairy industries (Munekata et al., 2022). It is now well known that a careful selection and use of autochthonous microbial strains can contribute to obtaining high-quality, functional, and safe dairy products (Gómez et al., 2016; Campagnollo et al., 2018; Fusco et al., 2019; Ait Chait et al., 2021; Huang et al., 2021). Such strains are mostly isolated from traditionally made cheeses or raw milk (Carafa et al., 2015; Terzić-Vidojević et al., 2015; Frau et al., 2016; Fusco et al., 2019; Özkan et al., 2021).

Many studies have focused on the genetic and functional diversity of *Streptococcus thermophilus* strains (Giraffa et al., 2001; Morandi and Brasca, 2012; Vendramin et al., 2017; Alexandraki et al., 2019), but few have studied the genetic diversity of wild strains of this species (Andrighetto et al., 2002; Erkus et al., 2014). Few studies have been conducted on the biodiversity analysis of *Streptococcus thermophilus* strains using whole genome sequencing (Hu et al., 2020). All these studies reported high genetic and technological diversity within *S. thermophilus* strains isolated from dairy products.

Protected designation of origin (PDO) cheese producers from Massif Central (France) mostly use commercial starter cultures for the manufacture of cheeses. The use of commercial starters in cheese production results in the loss of typical characteristics of the products (Hansen, 2002). To strengthen the link with the area and improve the sensorial qualities and typicality of cheeses, PDO cheese producers are increasingly interested in the development of specific autochthonous starter cultures. To meet this demand, a first step would be to isolate new wild strains from the producing areas and investigate the genetic and functional diversity of these strains within these reservoirs. Saint-Nectaire is an uncooked pressed cheese that was awarded the PDO label in 1955. Made in one of the smallest PDO areas in Europe, between Puy-de-Dôme and Cantal (France), this is the third French PDO cheese with cow’s milk.

The present study explores the genetic and functional diversity of wild strains of *Streptococcus thermophilus* from natural habitats at a local scale. This diversity was explored in the Saint-Nectaire cheese-producing PDO area, one of the smallest PDO areas in Europe with only 1,800 km^2^. The comparative analysis of the whole genome sequences of 26 wild strains of *Streptococcus thermophilus* was carried out. Important functional properties of the isolates were investigated, including milk acidification and proteolytic activities.

## 2. Materials and methods

### 2.1. Isolation and identification of *Streptococcus thermophilus* strains

#### 2.1.1. Isolation of strains

Presumptive *Streptococcus thermophilus* strains were isolated from (i) 62 milk samples (heated for 6 h at 42°C) collected on 31 different farms in the Saint-Nectaire cheese-producing PDO area (France) in summer and in winter to study the seasonal effect on biodiversity and (ii) commercial starter cultures. Isolation was carried out on M17 agar medium (Biokar Diagnostics, Beauvais, France) incubated at 42°C for 48 h. Representative colonies were picked out of this medium, purified twice, and maintained frozen at −80°C in an M17 broth medium containing 20% (v/v) glycerol. One *Streptococcus thermophilus* isolate was obtained from a bacterial collection of LAB isolated in 1998 from Saint-Nectaire cheeses (INRAE-ARILAIT collection). The Saint-Nectaire PDO area spreads over 69 municipalities between Puy-de-Dôme and Cantal (France), which are referenced in Supplementary Table S1. The specifications for the PDO of Saint-Nectaire cheese describe the conditions for cow breeding, milk production, cheese manufacturing, and ripening of cheeses.^1^

#### 2.1.2. 16S rDNA identification

16S rDNA of Saint-Nectaire isolates was amplified with the universal primers WO2 (5′-100 GNTACCTTGTTACGACTT-3′) and W18 (5′-GAGTTTGATCMTGGCTCAG-3′), as described previously by Callon et al. (2004). Polymerase chain reaction (PCR) amplification was carried out in a final volume of 50 μL containing 1X PCR buffer with MgCl_2_, 1 colony of isolates, 0.25 mM each primer, 200 mM each dNTP, and 1 U Taq DNA polymerase. The thermal cycling conditions were 94°C for 3 min, followed by 25 cycles of 94°C for 30 s, 50°C for 30 s, 72°C for 1 min 30 s, and a final extension step of 72°C for 10 min. The 16S rDNA gene sequencing was performed by LGC Biosearch Technologies (Berlin, Germany).

16S rDNA of commercial strains was amplified with the universal primers A (5′-AGAGTTTGATCCTGGCTCAG-3′) and H (5′-AAGG AGGTGATCCAGCCGCA-3′; Feurer et al., 2004). PCR amplification was carried out as described above for the Saint-Nectaire isolates. The 16S rDNA gene sequencing was performed by Eurofins Genomics (Konstantz, Germany).

To identify the partial 16S rDNA sequences obtained, a search of the NCBI GenBank DNA database was conducted using the BLAST algorithm. The percentage of similarity with DNA sequences deposited in this bank was determined.

16S rDNA sequences were deposited in the NCBI GenBank database under accession numbers OR350537-OR350561.

#### 2.1.3. Selection of representative isolates

In total, 58 isolates and 4 commercial strains were identified as *Streptococcus thermophilus* and were screened for their acidifying and proteolytic activity and for their growth characteristics in sterilized skimmed milk. Notably, 200 mL of sterilized skimmed milk (LACTALIS Ingredients, Bourgbarré, France) was inoculated with isolates at 10^6^ cfu.mL^−1^ and incubated for 24 h in a temperature-controlled batch reactor programmed to simulate the decrease in temperature during the manufacture of Saint-Nectaire cheese type (decrease from 33°C to 9°C over 24 h). The acidification kinetics were studied by continuous pH recording (iCINAC system). Proteolysis abilities of isolates were measured using the OPA (o-phtalaldehyde) method described by Church et al. (1983). Growth characteristics of isolates were determined by enumeration of isolates on M17 agar medium every hour for 24 h.

Three groups of isolates were formed based on these results. A total of 22 isolates representative of these three groups were selected for further characterization in this study. All selected isolates collected from the farms were isolated from dairy farms with milk production and cheese manufacturing directly on the farm.

### 2.2. Genetic characterization of strains

#### 2.2.1. DNA extraction and sequencing

Genomic DNA was extracted from cell cultures with Nuclospin® Tissue from Macherey Nagel according to the manufacturer’s instructions. Final concentrations were measured with a Qubit™ fluorometer using the dsDNA Broad Range Kit (Thermo Fisher Scientific). The extracted DNA was further sequenced using Illumina technology. Library preparation and sequencing were handled by Eurofins genomics (Konstantz, Germany) using a Novaseq 6000 sequencing system (Illumina, San Diego, CA, United States).

#### 2.2.2. Genome assembly and annotation

Sequencing reads from raw fastq files were filtered at Q30 with a minimal length of 110 bp with prinSeq (Cantu et al., 2019). Reads with remaining sequencing adapters were excluded with cutadapt V4.1 (Martin, 2011). Each genome was assembled using Spades V3.13 (Bankevich et al., 2012) with the careful option and annotated with Bakta V1.5 (Schwengers et al., 2021). EBI accession numbers are referenced in Table 1.

**Table 1:** *Streptococcus thermophilus* isolation sources with genomes assembly accession numbers analyzed in this study.

Isolates	Isolation source	Season of isolation	EBI accession ID project
19M1a	Farm 19	Summer	GCA_950101965
19bM2	Farm 19	Summer	GCA_950102045
21bM2	Farm 21	Summer	GCA_950101935
26bM1	Farm 26	Summer	GCA_950102035
26bM3	Farm 26	Summer	GCA_950101995
29bM1	Farm 29	Summer	GCA_950101945
29bM2	Farm 29	Summer	GCA_950102105
H10bM5	Farm 10	Winter	GCA_950101905
H11M1a	Farm 11	Winter	GCA_950102095
H11M1b	Farm 11	Winter	GCA_950102065
H11M1c	Farm 11	Winter	GCA_950102075
H11bM1	Farm 11	Winter	GCA_950102085
H11bM2	Farm 11	Winter	GCA_950102055
H14bM5	Farm 14	Winter	GCA_950102025
H20bM1	Farm 20	Winter	GCA_950101925
H23bM1	Farm 23	Winter	GCA_950101975
H23bM2	Farm 23	Winter	GCA_950101915
H23bM3	Farm 23	Winter	GCA_950101895
H26bM2	Farm 26	Winter	GCA_950102015
H26bM3	Farm 26	Winter	GCA_950101955
H26M3c	Farm 26	Winter	GCA_950102005
M134	ARILAIT collection	–	GCA_950101985
CS1	Commercial starter	–	PRJEB61322
CS3	Commercial starter	–	PRJEB61322
CS4	Commercial starter	–	PRJEB61322
CS2	Commercial starter	–	PRJEB61322

#### 2.2.3. Pan-genome assembly and visualization

Annotated GFF3 files of 22 *Streptococcus thermophilus* isolated from the Saint-Nectaire cheese-producing PDO area and 4 strains of *Streptococcus thermophilus* isolated from commercial starter cultures genomes were submitted to Roary (Page et al., 2015) for pan-genome analysis using default parameters. A gene presence–absence data matrix was derived and visualized using Phandango (Hadfield et al., 2018).

#### 2.2.4. Phylogenetic reconstruction

Nucleotide sequences of 1,350 core genes (excluding duplicated genes) were extracted from Roary results. They have been aligned with Mafft (Katoh and Standley, 2013), and distance computing and tree sampling were realized with BEAST (Suchard et al., 2018). A strict molecular clock was chosen with the HKY substitution model. BEAST (Suchard et al., 2018) is a program for Bayesian analysis of molecular sequences using MCMC (Markov chain Monte Carlo). It is entirely orientated toward rooted, time-measured phylogenies inferred using strict or relaxed molecular clock models. BEAST uses MCMC to average over tree space, so that each tree is weighted proportional to its posterior probability. We chose the strict molecular clock model with the HKY substitution model because this assumes that all branches on the tree have the same rate of evolution. This appeared reasonable considering the absence of knowledge on those rates for our new isolates.

Core-genome single nuclear polymorphism (SNP) tree was created with Parsnp (Treangen et al., 2014) on the Galaxy platform (The Galaxy Community, 2022). The resulting phylogenetic tree was visualized using iTOL.^2^ Parsnp is a genome multi-alignment tool designed to align genome sequences. It aligns and provides the output as the multiple sequence alignment of given sequences, SNP variations, and the core genome phylogeny. For this reconstruction, LMD-9 was used as the reference genome. The genome sequencing of *Streptococcus thermophilus* LMD-9 was described previously (Makarova et al., 2006), and the complete genome sequence can be accessed at GenBank under accession number CP000419.

Kyoto Encyclopedia of Genes and Genomes (KEGG) numbers (Kanehisa and Goto, 2000; Kanehisa, 2019; Kanehisa et al., 2023) for accessory genes were obtained using the eggnog-mapper v2 web tool (Iyer et al., 2010). Partial least squares discriminant analysis (PLS-DA) was performed using the mixOmics package of R software V 4.2.3.^3^

### 2.3. Technological characterization of isolates

#### 2.3.1. Preparation of model cheese curd

The model cheese curds (MCC) were prepared according to Callon et al. (2016) with some modifications. In total, 40 mL of pasteurized milk (Ferme des Peupliers, Normandie, France) was incubated at 33°C and inoculated with isolates at 10^6^ cfu.mL^−1^. The milk was coagulated with 12 μL of calf rennet for 45 min at 33°C and then centrifuged for 20 min at 20,000 *g* at 33°C. The supernatant (lactoserum) was discarded, and the curd was incubated for 24 h in a temperature-controlled batch reactor programmed to simulate the decrease in temperature during the manufacture of Saint-Nectaire cheese type (decrease from 33°C to 9°C over 24 h). Each strain was tested three times.

#### 2.3.2. Growth characteristics

To determine the growth ability of isolates, MCC samples were collected at 0, 6, and 24 h. MCC samples were blended two times for 2 min in a phosphate buffer of pH 7.5 using a Stomacher blender (Interscience, St Nom la Bretèche, France) for dissociating all the cell aggregates. After appropriate dilution in Ringer’s solution, *S. thermophilus* isolates were enumerated on M17 agar and incubated at 30°C for 48 h.

#### 2.3.3. Acidifying activity

To analyze the acidifying activities of isolates, MCC samples were collected at 0, 6, and 24 h, and pH was measured in the core of the MCC samples using a WTW pH 526 pH meter with a LOT406-M6-DXK electrode (Mettler-Toledo S.A, Viroflay, France). A classification of isolates based on their acidifying activities was constructed according to that previously reported by Morandi and Brasca (2012), with some modifications. The pH unit target values were modified because we worked on a model cheese curd with a temperature decrease mimicking Saint-Nectaire cheesemaking (33°C to 9°C), while precedent studies worked on fermented milk at a constant temperature of 37°C.

#### 2.3.4. Proteolytic activity

The extraction of water-soluble nitrogen from the MCC samples was carried out according to the method described by Myagkonosov et al. (2021) with some modifications. Five grams of MCC samples were mixed with 5 cm^3^ of deionized water and homogenized with a stomacher for 4 min. The resulting mixture was transferred to a volumetric flask, and the volume was made up to 100 mL with deionized water. The mixture was kept at 40°C for 1 h with continuous shaking. The samples were centrifuged at 3,000 *g* for 30 min. After centrifugation, the samples were cooled to 4°C, and the upper fat layer was removed. The supernatant was separated and filtered with a pore size of 0.45 μm. The resulting filtrate was mixed with deionized water at a ratio of 1:5. Next, 3 mL of OPA (o-phtalaldehyde) reagent prepared according to Church et al. (1983) was added to 300 μL of the solution, and after 2 min, absorbance was measured at 340 nm with a 7,200 spectrophotometer Jenway (Dutscher, France). The results have been expressed in mmol.L^−1^ of glycine based on a calibration curve. The proteolytic activity of isolates was determined at 24 h of fermentation by subtracting the results at 0 h of fermentation.

#### 2.3.5. Statistical analysis

Statistical analysis of biochemical data was performed using the XLSTAT software (Addinsoft, Paris, France). The results are reported as means ± standard deviation. The normality of the data was checked using a Shapiro–Wilk test (*p* < 0.05). The test rejected the hypothesis Ha of normality when the value of *p* was ≤0.05. A large number of variables did not have a normal distribution, and therefore a non-parametric test (Kruskal-Wallis) and *post-hoc* comparison (Conover-Iman procedure) were used to compare the concentrations obtained. Differences between the mean values were considered significant at *p* < 0.05. Pearson’s correlations at the 5% significance level (*p* < 0.05) were used to explain the relationship between functional properties.

## 3. Results and discussion

### 3.1. Isolation and identification

In this study, strains belonging to the *Streptococcus thermophilus* species were isolated from the Saint-Nectaire cheese-producing PDO area (France).

All isolates were identified on the basis of 16S rDNA sequence alignment using the NCBI blast algorithm. Isolates showing a percentage of similarity equal to or higher than 99% with *Streptococcus thermophilus* DNA sequences available in this database were considered to be *Streptococcus thermophilus*. In total, 58 isolates were identified as *Streptococcus thermophilus*.

Twenty-two isolates collected throughout the geographical producing area and representative of diversity, together with four commercial strains, were selected and characterized for their genetic and technological properties. Conditions of isolation and the EBI accession number are referenced in Table 1. The genome sequencing and assembly-related information are shown in Supplementary Table S2.

### 3.2. Genetic diversity

#### 3.2.1. Phylogenetic reconstruction

**Figure 1:**
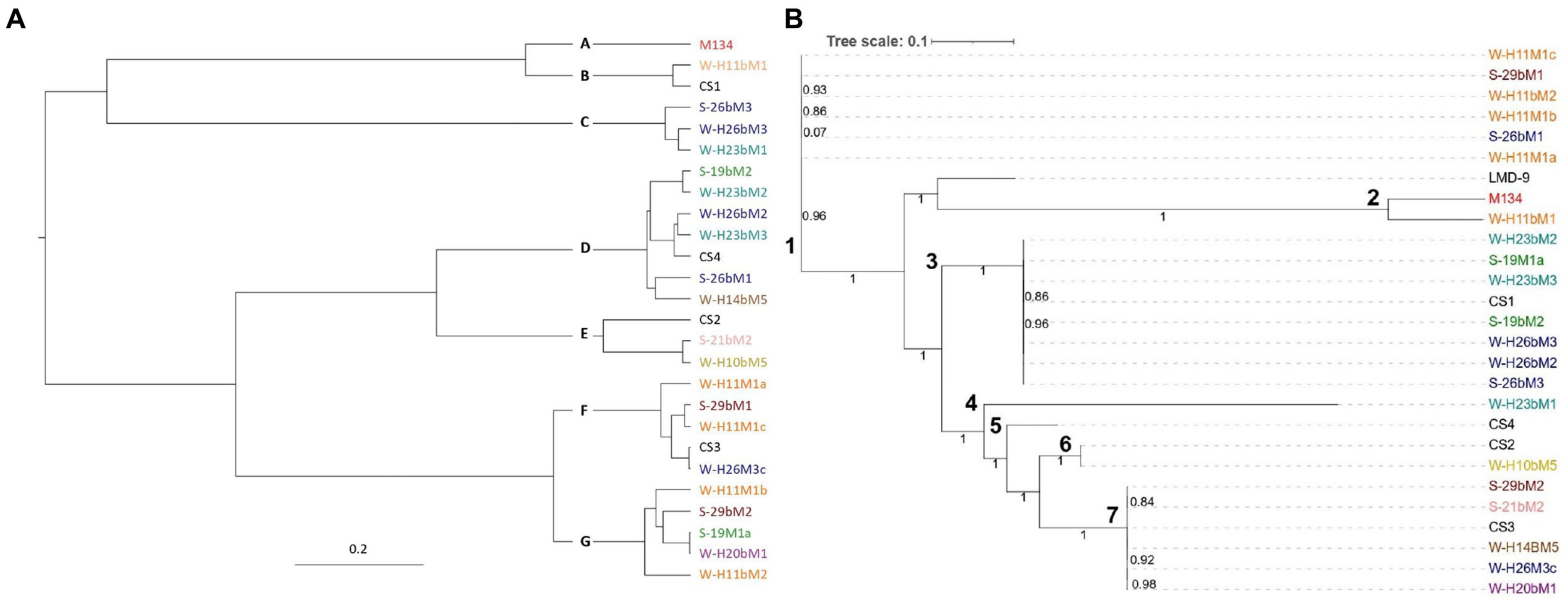
Phylogenetic trees of the 26 *Streptococcus thermophilus* isolates based on **(A)** core genome and **(B)** single nucleotide polymorphisms. Isolates from summer milks are annotated with an “S” and isolates from winter milks are annotated with a “W.” Isolate names with the same colors were isolated from the same farm.

Two phylogenetic analyses were performed (Figure 1). The first one was based on core-gene alignment using the Bayesian method and allowed to identify seven clades (Figure 1A), namely, A–G. The other was based on single nucleotide polymorphisms (SNPs) detected (Figure 1B) and allowed the distinction of seven clades, namely, 1–7.

From the two resulting trees, it could be noticed that SN-isolates from the same farms were distributed in various clades. For example, in Figure 1A, isolates H11M1a, H11bM1, and H11M1b were all isolated on farm 26, but were not located in the nearby clade. A similar situation is observed in Figure 1B for isolates 26bM1, 26bM3, and H26M3c. Moreover, strains from different farms may be clustered together.

These results indicate that the phylogenetic reconstruction of isolates did not correspond to their geographical distribution, despite a small isolation area (1,800 km^2^) and closed milk production. Vendramin et al. (2017) and Hu et al. (2020) reported similar results with strains isolated from China and Italy, respectively, compared to strains isolated from other continents. This distinction indicated that the isolation source did not exert an important impact on the evolution of *Streptococcus thermophilus*, although the farms are quite close to one another. It is also notable that isolates from summer and winter milk were distributed into different clades and not clustered together, suggesting that the genetic variability of the isolates did not depend on the season of isolation.

#### 3.2.2. Pan-genome analysis

The pan-genome of the 22 Saint-Nectaire isolates and the 4 commercial strains was performed by clustering the genes encoding complete protein sequences into core and accessory genomes using Roary (Page et al., 2015).

**Figure 2:**
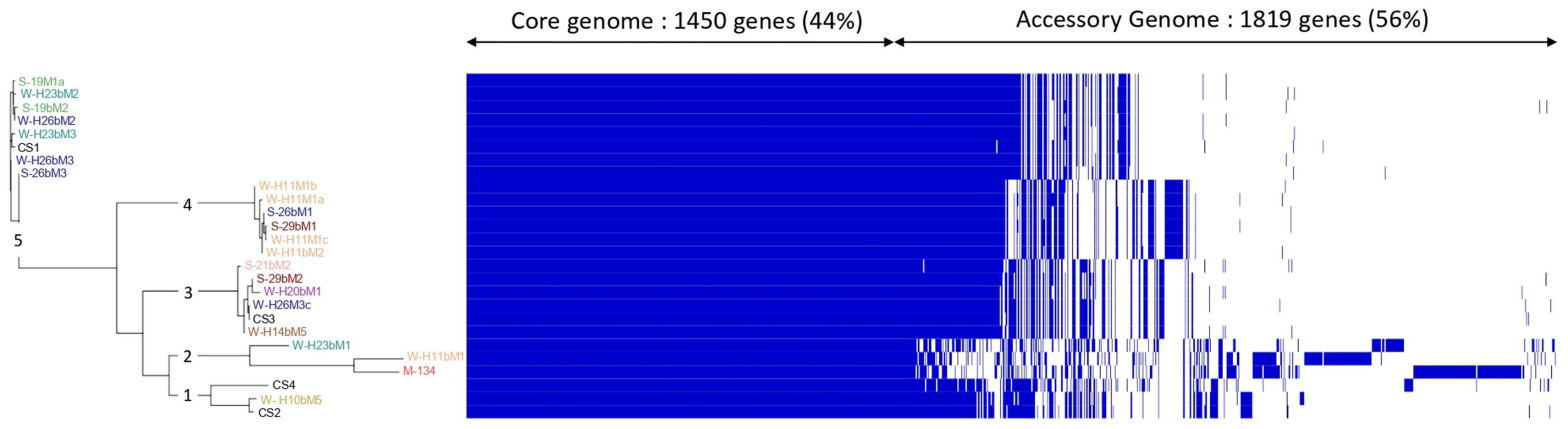
Clustering of isolates associated with the visualization of the *Streptococcus thermophilus* isolate pan-genome. The pan-genome was visualized based on the Phandandgo software (Hadfield et al., 2018). In the Roary matrix, genomes are shown as rows, and homologous gene clusters are depicted as columns. The presence of a gene cluster in a genome is indicated by blue. Core gene clusters that are found in all genomes are shown on the left side of the matrix. Isolates from summer milks are annotated with an “S” and isolates from winter milks are annotated with a “W.” Isolate names with the same colors were isolated from the same farm.

The core genome is the set of genes shared by all the genomes studied and can be divided into hard-core genes, which are defined to be present in >99% of the genomes, and soft-core genes, which are present in 95%–99% of the genomes. The accessory genome is shared by a subset of the genomes tested and is subdivided into shell genes, which are present in 15%–95% of genomes, and cloud genes, which are found in less than 15% of genomes. From the 3,269 genes constituting the pan-genome of the 26 *Streptococcus thermophilus* isolates studied here, we found 1,450 (44%) core genes (present in 24/26 genomes), including 1,355 (41%) hard core genes (present in 25/26 genomes) and 95 (3%) soft core genes (present in 24/25 genomes), and 1,819 (56%) accessory genes (present in less than 24 genomes), which might be responsible for the fundamental differences in phenotypic characteristics between the isolates (Daughtry et al., 2018), including 776 (24%) shell genes (present in 3–24 genomes) and 1,053 (32%) cloud genes (present in less than 3 genomes; Figure 2). These results are similar to those reported by Alexandraki et al. (2019), who analyzed the pan-genome of 23 strains of *S. thermophilus* and identified 1,089 core genes (52%) and 997 accessory genes (48%).

The generated clustering based on the pan-genome was concordant with the phylogenetic reconstruction based on SNPs (Figure 1B) in defining the relationships among isolates. It showed five main clusters with obvious differences in accessory genes for each of them (Figure 2). The first cluster consisted of SN-isolate H10bM5 and commercial strains CS4 and CS2. Cluster 2 included SN-isolates H11bM1, M134, and H23bM1. SN-isolates 21bM2, 29bM2, H20bM1, H26M3c, H14bM5, and commercial strain CS3 were clustered together. The fourth cluster comprised SN-isolates H11M1b, H11M1a, 26bM1, 29bM1, H11M1c, and H11bM2. Finally, the fifth cluster consisted of SN-isolates 19M1a, H23bM2, 19bM2, H26bM2, H23bM3, H26bM3, 26bM3, and commercial strain CS1. These results reveal a high genetic variation level probably related to the accessory gene content across isolates, which are involved in different functional categories. These findings are in accordance with Giraffa et al. (2001), Andrighetto et al. (2002), and Morandi and Brasca (2012), who also reported a high genetic variability of *Streptococcus thermophilus* with the RAPD-PCR method.

#### 3.2.3. KEGG functional analysis

**Figure 3:**
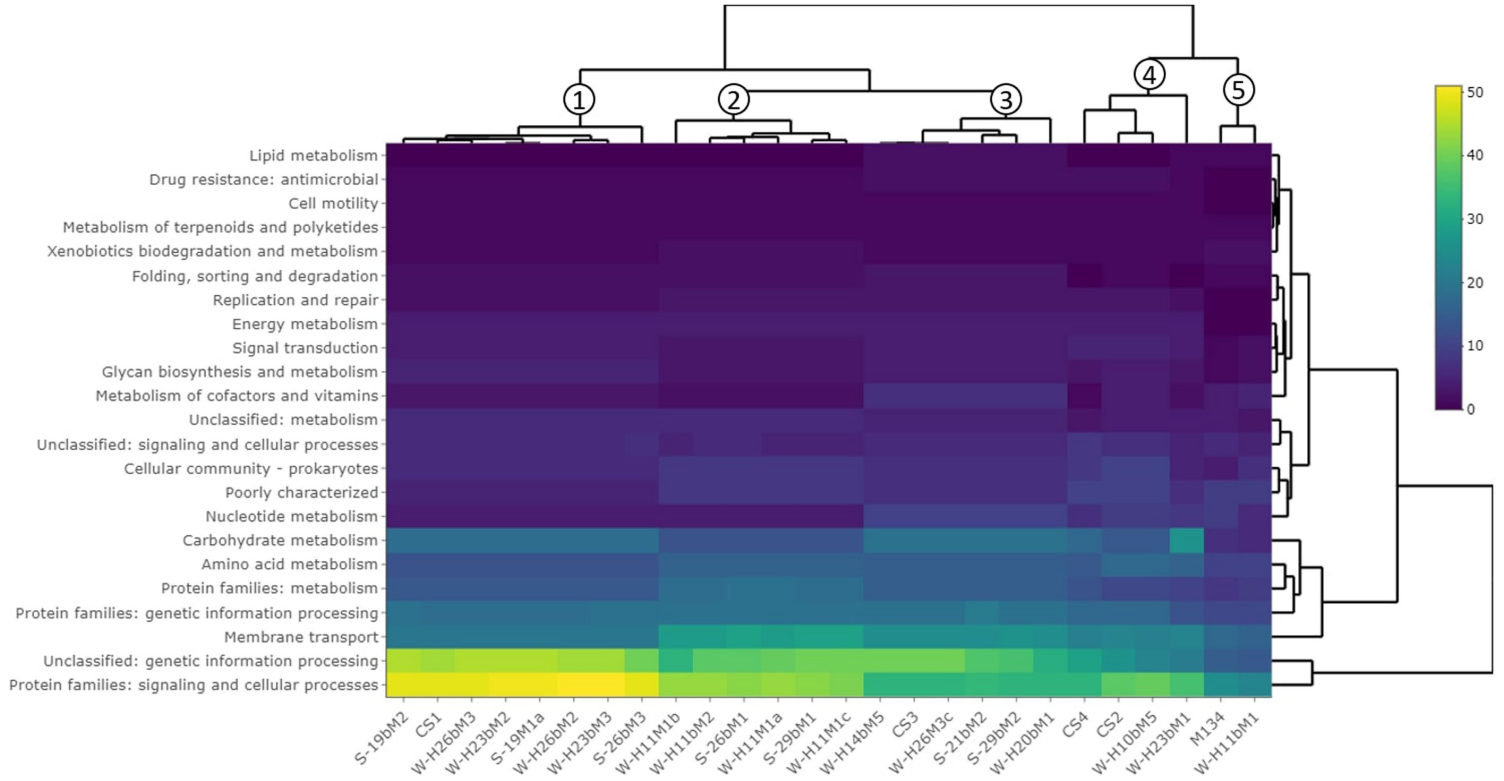
Hierarchical clustering of isolates based on the gene content summarized by KEGG pathways. The clustering on top of the figure shows similarities between isolates.

A hierarchical clustering of the isolates was performed on the accessory genes annotated with the KEGG pathway (Figure 3). Almost 77% of the accessory genes were positively annotated with a KEGG ko number. These genes were divided into 23 functional KEGG pathways, the most important being “Protein families: signaling and cellular processes” (18% of the total annotated genes), “Unclassified: genetic information processing” (16%), “Membrane transport” (10%), “Carbohydrate metabolism” (7%), “Protein families: genetic information processing” (7%), “Protein families: metabolism” (6%), and amino acid metabolism (6%). These results are in accordance with Vendramin et al. (2017), who performed a genome comparison of eight *Streptococcus thermophilus* strains of dairy origin isolated in Italy. They investigated the strain-specific features by assigning functional categories of the SEED subsystem to the gene content of the strains. Among the functional categories identified, four accounted for a large part of the strain diversity, including “amino acids and derivatives,” “carbohydrates,” “DNA metabolism,” and “membrane transport,” covering almost 50% of the specific genes. Although the annotations are different due to the databases used, their results are similar to the present study.

This hierarchical clustering (Figure 3) allowed an evaluation of the similarity between isolates based on the number of accessory genes associated with a functional KEGG pathway. Five groups were identified and are similar to those observed in the phylogenetic tree based on the pan-genome with a difference (Figure 2).

The first group is composed of isolates H23bM2, 19bM2, 19M1a, H26bM3, 26bM3, and CS1. The second group includes isolates H11M1c, 29bM1, H11M1a, 26bM1, H11bM2, and H11M1b. Isolates 21bM2, H14bM5, H26M3c, 29bM2, H20bM1, and CS3 formed the third group. Isolates H10bM5, H23bM1, CS4, and CS2 were clustered in the fourth group, and the fifth group consisted of isolates M134 and H11bM1.

**Figure 4:**
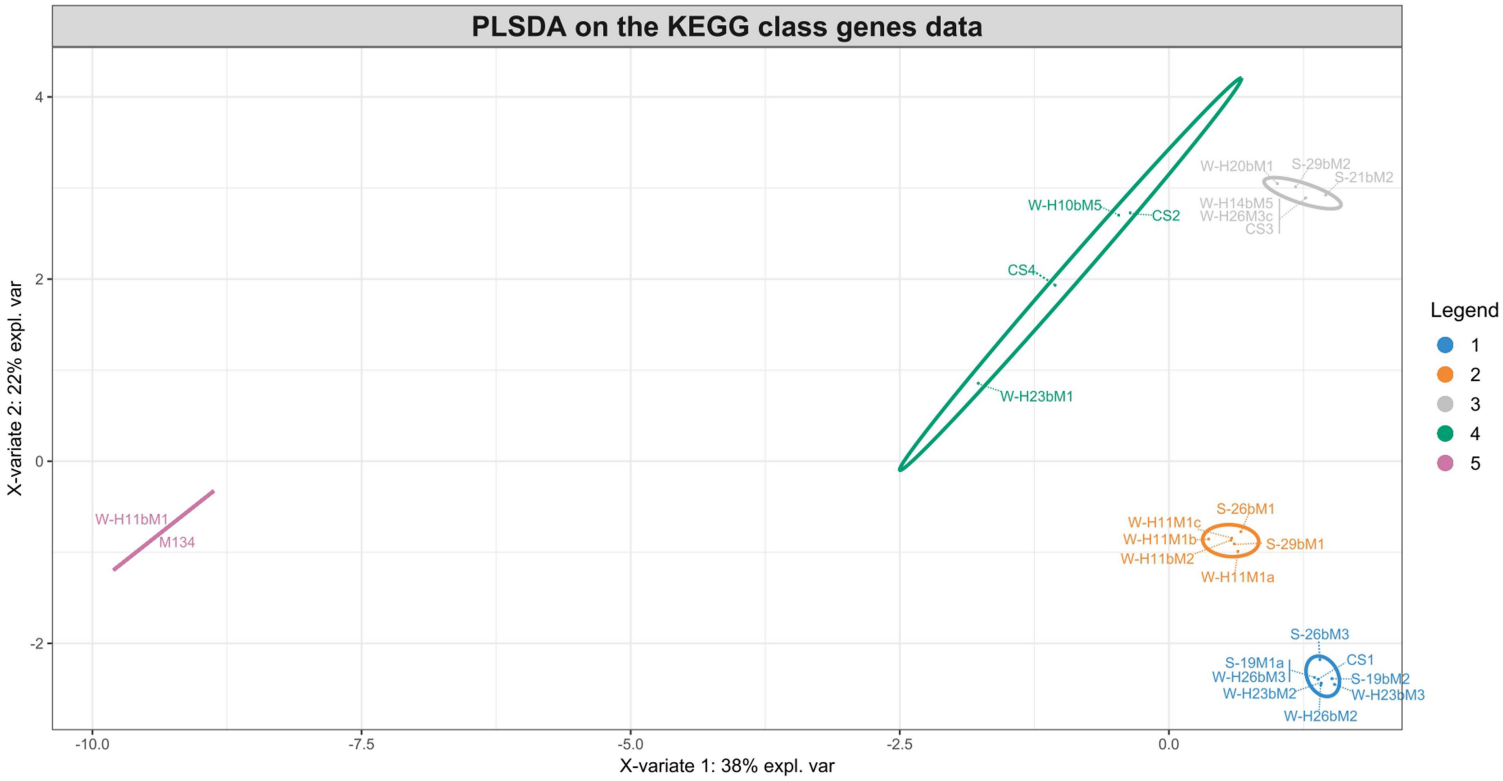
Sample plots from the PLS-DA analysis performed on accessory gene numbers of the KEGG class for groups 1 (blue), 2 (orange), 3 (gray), 4 (green), and 5 (pink).

**Figure 5:**
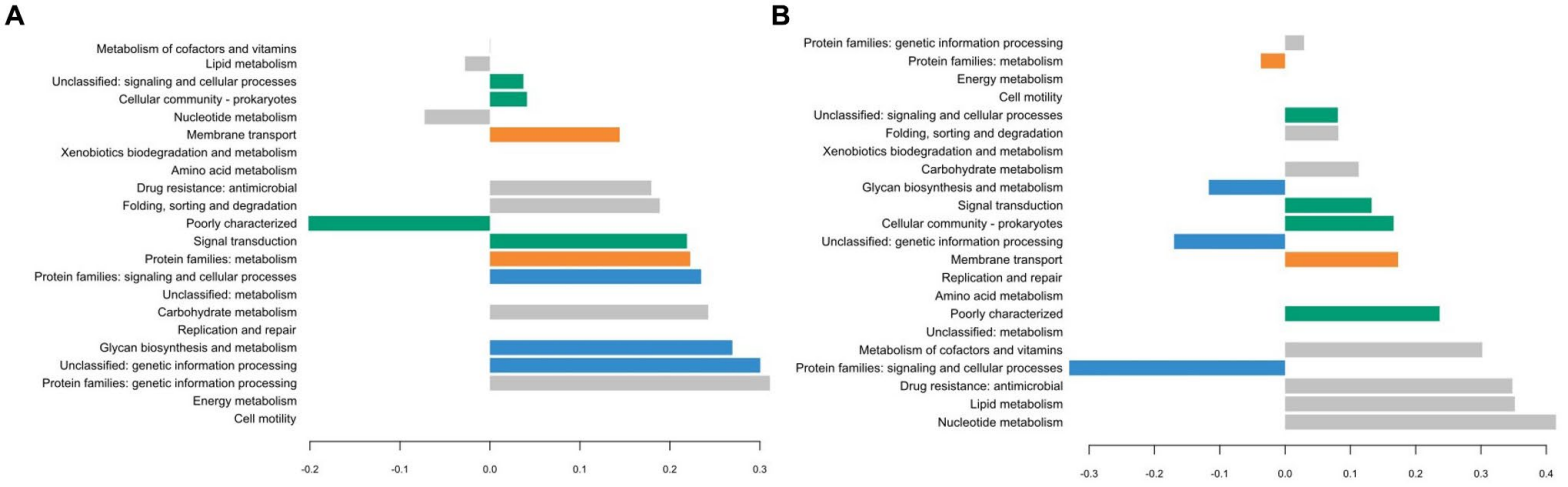
Plot loadings representing the variable’s contribution on component 1 **(A)** and on component 2 **(B)** of the PLS-DA analysis. Group 1 is colored in blue, 2 in orange, 3 in gray, 4 in green, and 5 in pink.

**Figure 6:**
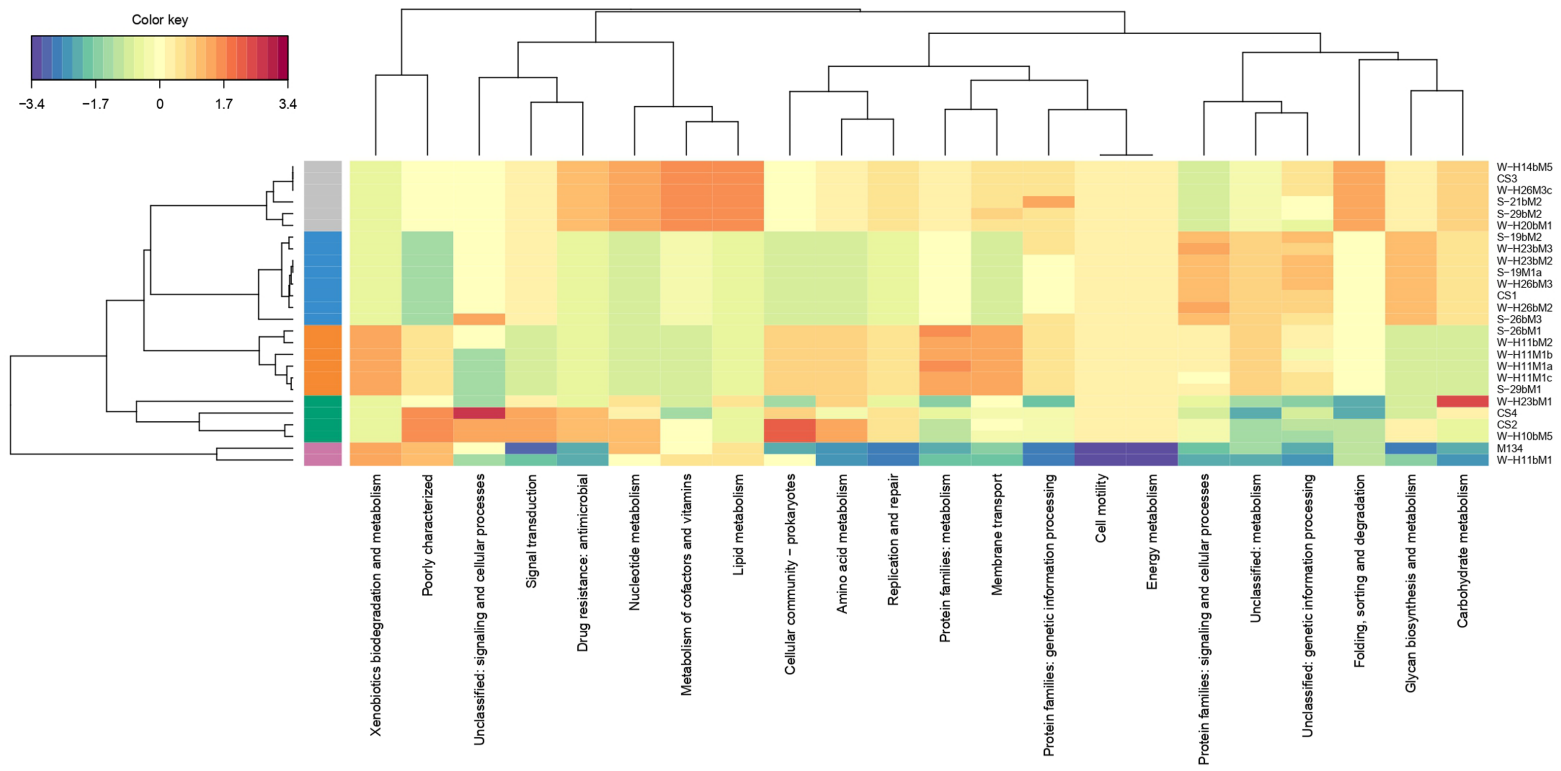
Clustered image map (CIM) associated with PLS-DA analysis. Groups are distinguished by colors on the left side of the map. Group 1 is colored in blue, 2 in orange, 3 in gray, 4 in green, and 5 in pink.

The different groups stand out for their functional KEGG class. A PLS-DA analysis was performed (Figure 4) to identify KEGG classes that differentiate isolates into these groups. PLS-DA results showed that the groups were easily recognized based on the accessory gene content of each KEGG class. These KEGG classes may be the indicators to distinguish these three clades. Figure 5 shows the variable’s contribution from components 1 (A) and 2 (B) of the PLS-DA analysis, and Figure 6 represents the clustered image map (CIM) associated with it. These figures showed the functional KEGG pathways overrepresented in each of the clades that allowed their differentiation. Heatmaps, representing the lowest level of the KEGG pathway database (gene ko level), were constructed for each KEGG pathway identified as characteristic of a group (Supplementary Figures S1–S4).

The first group was characterized by three KEGG pathways, namely, “Protein families: signaling and cellular process,” “Unclassified: genetic information processing,” and “Glycan biosynthesis and metabolism.” More accessory genes encoded for transposases and putative transposases were identified in the “Unclassified: genetic information processing” KEGG pathway for the first group in comparison with others (Supplementary Figure S1). Transposases are typically present within bacterial genomes as small, mobile elements solely comprising their own transposition genes; they are important factors for horizontal gene transfer between strain genomes and are therefore important for the safety evaluation of strains for their use as starter cultures. However, many of the accessory genes of the first group, grouping in the “Protein families: signaling and cellular processes,” were involved in the clustered regularly interspaced short palindromic repeat (CRISPR) and CRISPR-associated (Cas) proteins immune defense system in comparison to other groups (Supplementary Figure S2). It is now well known that the CRISPR/Cas system confers resistance against foreign genetic elements, such as phages (Bolotin et al., 2004; Mojica et al., 2005; Lillestøl et al., 2006). As phage infection of starters is the most common cause of incomplete fermentation in the dairy industry, such genes could represent a great advantage for the use of these isolates as starter cultures.

Then, the KEGG pathways “Protein families: metabolism” and “Membrane transport” allowed the differentiation of the second group, which might confer benefits to isolates during the manufacture of cheeses since accessory genes associated with these functional KEGG pathways encoded for dipeptidases (pepDA, pepDB; Supplementary Figure S3) and peptide transport systems (oligopeptide transport system Opp; Supplementary Figure S4), among other things, which are involved in proteolysis and thus are important for aromatic and textural properties of cheeses (Savijoki et al., 2006; Rodríguez-Serrano et al., 2018).

The third group stands out for the KEGG pathways “Protein families: genetic information processing,” “Nucleotide metabolism,” “Metabolism of cofactors and vitamins,” “Drug resistance: antimicrobial,” “Folding, sorting, and degradation,” and important functional categories for cheese manufacturing, including “carbohydrate metabolism” and “Lipid metabolism.” Carbohydrate metabolism is the most important metabolism pathway for starter cultures because of their primary role in producing lactic acid from lactose (Hutkins and Goh, 2014). Lipid metabolism is also of high interest because lipolysis plays an essential role in the development of flavors in cheeses (McSweeney and Sousa, 2000).

The KEGG pathways “Cellular community: prokaryotes,” “Signal transduction,” and “Unclassified: signaling and cellular process” characterized the fourth group and might facilitate the survival of cells under stressful environmental conditions. These functional traits may be of great advantage as the manufacture of cheeses exposes starters to various environmental stresses such as low pH, osmotic stress, and high pressure (Zotta et al., 2008).

The PLS-DA analysis did not identify the characteristic KEGG functional class of the fifth group, probably due to their lower content of accessory genes in comparison with other isolates, with only 210 accessory genes for isolate M134 and 207 for isolate H11bM1. However, these isolates were distantly separated from commercial strains based on the phylogenetic reconstruction based on core genes (Figure 1) and on the clustering associated with the pan-genome (Figure 2), which could be an added value for potential use as autochthonous starter cultures to strengthen the typical characteristics of cheeses.

Interestingly, commercial strains CS1, CS2, and CS3 were not significantly distantly separated from the centroid of their respective clusters, unlike the CS4 strain (Figure 2), indicating that commercial strains are representative of *Streptococcus thermophilus* genetic diversity. According to the PLS-DA analysis (Figures 4–6), the main differences between CS4 and SN-isolates H10bM5 and CS2 were the number of genes implicated in several KEGG categories, including “Protein families: metabolism” and “Carbohydrate metabolism.” In these categories, isolate CS4 had a higher number of accessory genes encoding dipeptidases (Supplementary Figure S3) and a higher content of accessory genes implicated in carbohydrate transport and metabolism (Supplementary Figure S5) than CS2 and H10bM5 isolates. However, these results focused only on accessory genes. To avoid this bias and study the carbohydrate metabolism of each isolate, a search for specific genes in their whole genomes was performed.

#### 3.2.4. Carbohydrate metabolism and sugar transport system

The sugar utilization ability of *S. thermophilus* strains is of major importance in dairy fermentation as it directly affects the rate of milk acidification. Various studies suggest that the utilization of glucose, lactose, and fructose by *S. thermophilus* is consistently observed, whereas the utilization of sucrose, galactose, mannose, and maltose exhibits variable profiles (O’Leary and Woychik, 1976; Thomas et al., 2011; Erkus et al., 2014).

**Figure 7:**
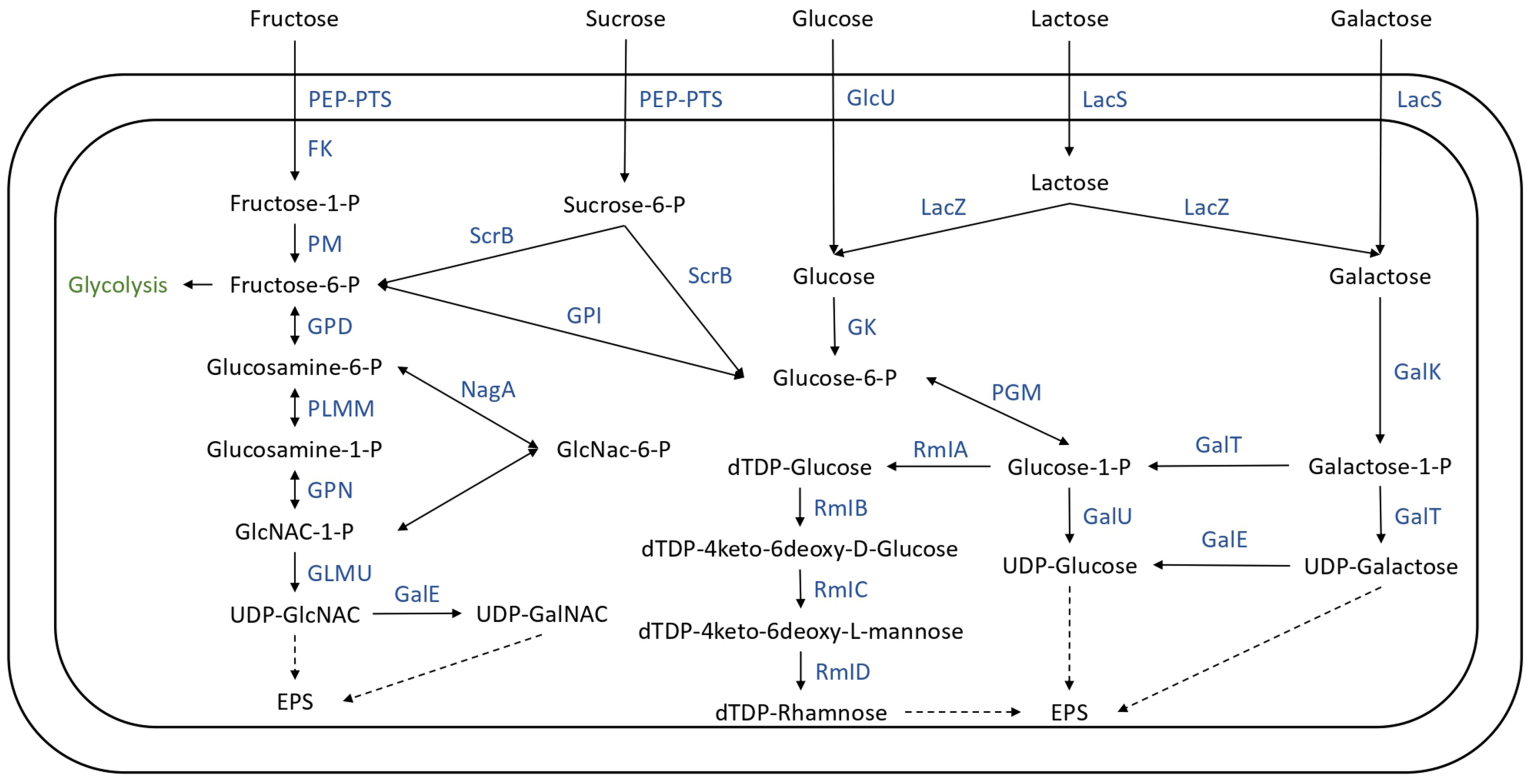
Carbohydrate transport and metabolism in the 26 *Streptococcus thermophilus* isolates. FK, fructokinase; GalE, UDP-galactose-4-epimerase; GalK, galactokinase; GalT, UTP-galactose-1-phosphate uridyltransferase; GalU, UDP-glucose pyrophosphorylase; GK, glucokinase; GlcU, glucose permease; GLMU, *N*-acetylglucosamine-1-phosphate uridyltransferase; GPD, glucosamine-6-phosphate deaminase; GPI, glucose-6-phosphate isomerase; GPN, glucosamine-1-phosphate *N*-acetyltransferase; LacS, lactose permease; LacZ, beta-glucosidase; Nag, *N*-acetyl-alpha-D-galactosaminidase; PEP-PTS, phosphoenolpyruvate-sugar phosphotransferase system; PGM, α-phosphoglucomutase; PLMM, phosphoglucosamine mutase; PM, phosphomutase; RMIa, glucose-1-phosphate thimidyltransferase; RmIb, dTDP-glucose 4, 6 dehydratase; RmIC, dTDP-4-deshydrorhamnose 3,5-epimerase; RmID, DTDP-4-keto-L-rhamnose reductase; ScrB, sucrose-6-phosphate hydrolase.

The lactose, glucose, galactose, sucrose, and fructose transport and utilization systems were analyzed in the genomes of the 22 SN-isolates and the 4 commercial strains (Figure 7), and the genes of each isolate implicated in these systems are listed in Supplementary Table S3.

Lactose represents the principal carbohydrate in milk and is the preferred carbon and energy source of *Streptococcus thermophilus* (Xiong et al., 2019), probably due to the adaptation of the species to the milk (Goh et al., 2011; Alexandraki et al., 2019). In this species, lactose transport and hydrolysis are controlled by the lac operon. It encodes a lactose permease (LacS) and a cytoplasmic β-galactosidase (LacZ) which cleave the lactose into glucose and galactose (Xiong et al., 2019). The 26 genomes all harbored the LacZ and LacS genes (Figure 7; Supplementary Table S3). The resulting glucose moiety is phosphorylated to glucose-6-P by glucokinase and is further utilized through the glycolysis pathway (Figure 7). The Leloir pathway is the most common route for galactose utilization in *S. thermophilus*. Galactose is converted to glucose-1-phosphate by the galRKTEM gene cluster, which consists of the regulator GalR, galactokinase (GalK), galactose-1-phosphate uridyltransferase (GalT), UDP-glucose-4-epimerase (GalE), and galactosemutarotase (GalM; Xiong et al., 2019; Figure 7). However, most strains of *S. thermophilus* are unable to ferment galactose despite having intact galRKTEM gene clusters for the Leloir pathway (Vaughan et al., 2001; de Vin et al., 2005). The most probable explanation for this observed phenotype is a deficient promoter leading to insufficient transcription (Vaughan et al., 2001).

Gal-positive strains are of technological importance principally for their ability to completely ferment lactose, which results in a reduced amount of galactose being present, which cannot serve as a carbon source for spoilage or pathogenic bacteria (Hutkins and Morris, 1987).

The DNA fragments of the *galR-galK* intergenic region of the 26 isolates were picked out from their genome sequences (Supplementary Figure S6). Four different types of fragments were detected in the 26 genomes analyzed. Isolates 19M1a, H23bM2, 19bM2, 26bM3, H23bM3, H26bM2, H26bM3, and CS1 are type A; 21bM2, 29bM2, H20bM1, H26M3c, CS3, H14bM5, H10bM5, CS2, CS4, and H23bM1 are type B; H11M1a, H11M1b, H11M1c, 26bM1, 29bM1, and H11bM2 are type C; and isolates M134 and H11bM1 belong to type D.

It is now well documented that a single point of mutation with a G-to-A substitution in the −9 position of the −10 box in the *galK* promoter results in improved *galK*-promoter activity and could be responsible for the ability to ferment galactose (Vaughan et al., 2001; van den Bogaard et al., 2004; de Vin et al., 2005; Giaretta et al., 2018; Xiong et al., 2019). The presence of this mutation was examined in the 26 genomes studied here. Consequently, among the isolates, only M134 and H11bM1 appeared capable of galactose utilization as they owned the relevant mutation related to the Gal-positive phenotype. However, de Vin et al. (2005) and Hu et al. (2020) demonstrated that the gal promoter does not exclusively determine the gal-positive phenotype of *Streptococcus thermophilus* strains. Experimental verification is therefore required to validate this prediction.

The utilization of sucrose and fructose by *S. thermophilus* required a phosphoenolyruvate-phosphotransferase system (PEP-PTS), consisting of a PEP-dependent phosphotransferase (enzyme I, EI), a histidine-containing phosphocarrier protein (HPr), and a sugar-specific permease (enzyme II, EII). The 26 genomes investigated all harbored a PEP-dependent phosphotransferase and the HPr, as well as genes responsible for sucrose and fructose PTS transporter sugar-specific permease enzymes (Figure 7; Supplementary Table S3). Furthermore, genes responsible for sucrose and fructose utilization detailed in Figure 7 were detected in these genomes (Supplementary Table S3). However, experimental procedures are required to identify the ability of these isolates to ferment these carbohydrates.

### 3.3. Technological diversity

**Figure 8:**
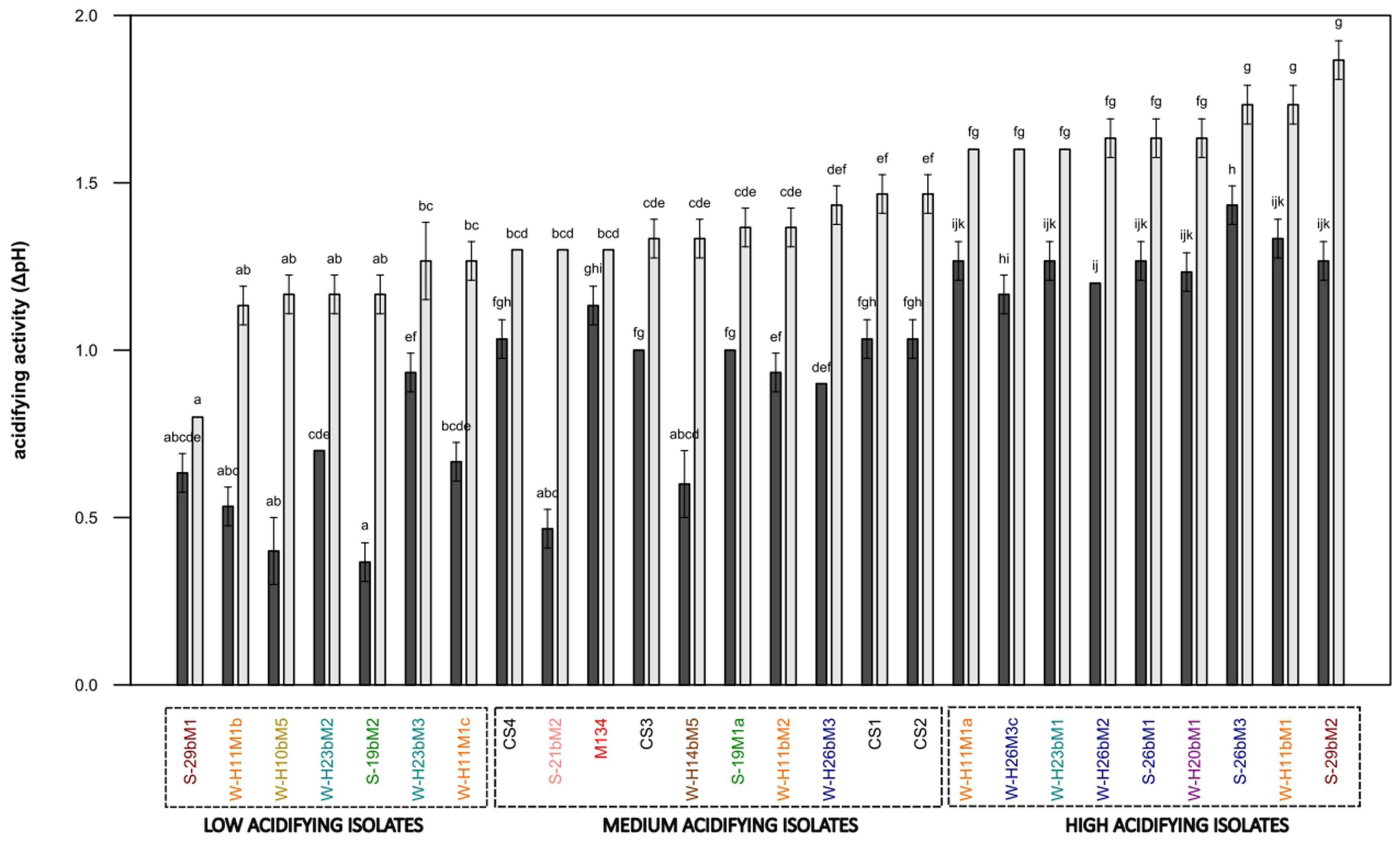
Acidifying activity of *Streptococcus thermophilus* isolates (a–k): mean values of the same time of fermentation without a common superscript are significantly different (*p* < 0.05) according to the Conover-Iman test. Isolates from summer milks are annotated with an “S” and isolates from winter milks are annotated with a “W.” Isolate names with the same colors were isolated from the same farm.

The acidifying abilities of 22 *Streptococcus thermophilus* isolated from the Saint-Nectaire cheese-producing PDO area (SN-isolates) and 4 strains of *Streptococcus thermophilus* isolated from commercial starter cultures were evaluated in model cheese curd. From the pH values obtained, ΔpH6h and ΔpH24h were calculated (Figure 8).

Half of the 22 SN-isolates lowered the pH by more than one pH unit after 6 h of fermentation, suggesting fast acidifying ability. As expected, all commercial strains presented fast acidifying abilities, which is one of the most important criteria for cheese manufacturing (Tidona et al., 2020).

Concerning the ability to reduce the pH of the model cheese curds at 24 h, the following three main groups of isolates were distinguished: (i) low acidifying isolates with a pH decrease below 1.30 pH units, including 32% of the SN-isolates (19bM2, H10bM5, H11M1b, H11M1c, H23bM2, H23bM3, and 29bM1); (ii) medium acidifying isolates, showing a pH decrease between 1.30 and 1.50 pH units, comprising 27% of total SN-isolates and 100% of the commercial strains (CS1, 19M1a, 21bM2, H11bM2, H14bM5, H26bM3, M134, CS3, CS4, and CS2); (iii) high acidifying isolates, causing a pH decrease higher than 1.50 pH units, regrouping 41% of the SN-isolates (H11M1a, H26bM2, H26M3c, 26bM1, 26bM3, 29bM2, H11bM1, H20bM1, and H23bM1). It could be noticed that SN-isolates from the same farms and SN-isolates from summer and winter milk are distributed into the three acidifying activity categories, and that the four commercial strains were clustered in the group of medium acidifying activity, while the autochthonous SN-isolates showed various acidifying activities.

This clustering reveals a high variability in acidifying activity observed within *Streptococcus thermophilus* SN-isolates, suggesting that this feature is strain-dependent, in accordance with Giraffa et al. (2001). This diversity of acidifying activities of the SN-isolates does not depend on the season or the farms. In contrast, commercial strains showed homogeneous acidifying activity. This homogeneity is probably due to the specific selection of the strains based on their functional characteristics to be used as starter cultures (Gibbons and Rinker, 2015).

**Figure 9:**
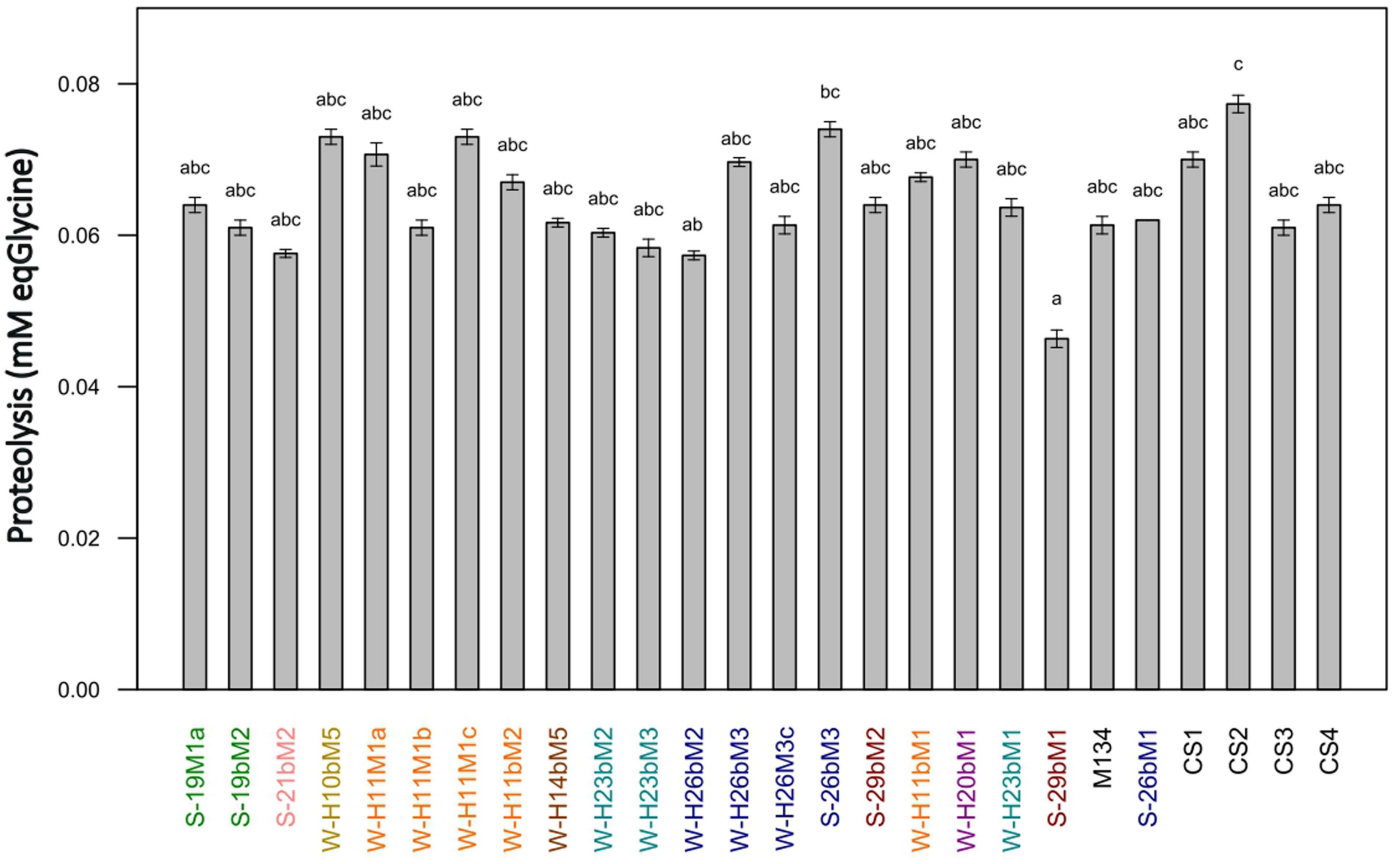
Proteolytic activities of *Streptococcus thermophilus* isolates (a–c): mean values without a common superscript are significantly different (*p* < 0.05) according to the Conover-Iman test. Isolates from summer milks are annotated with an “S” and isolates from winter milks are annotated with a “W.” Isolate names with the same colors were isolated from the same farm.

The proteolytic activities of the 22 SN-isolates and the 4 commercial strains of *Streptococcus thermophilus* were evaluated in a model cheese curd at 24 h of fermentation, and the results are presented in Figure 9. The proteolytic activities of *Streptococcus thermophilus* SN-isolates and commercial strains varied slightly, from 0.046 to 0.077 mmol_eqGlycine_.L^−1^ for 29bM1 SN-isolates and CS2 commercial strains, respectively.

Galia et al. (2009) and Dandoy et al. (2011) have highlighted a link between high acidifying activities and the presence of an efficient proteolytic system, whereas our results showed low proteolytic activities and a limited diversity on this criteria in comparison with other studies on the characterization of *Streptococcus thermophilus* strains (Urshev et al., 2014; Hu et al., 2020; Tidona et al., 2020) or other species (Zaaraoui et al., 2021; Abarquero et al., 2022; Sugajski et al., 2022). These findings are in accordance with Harnett et al. (2022), who reported *Streptococcus thermophilus* as a LAB species generally considered poorly proteolytic. To ensure sufficient proteolytic activity in dairy products, *Streptococcus thermophilus* is often associated with other species for the development of starter cultures, such as *Lactobacillus delbrueckii* subsp. *bulgaricus.* The symbiotic growth of both species leads to protocooperation, inducing higher proteolytic and acidifying rates (Hervé-Jiménez, 2008).

In addition, a PLS-DA analysis was performed on the KEGG annotation of accessory genes with acidification groups (Supplementary Figure S7) and indicated that the KEGG annotations of accessory genes did not allow for distinguishing the acidification groups. The lack of diversity in proteolytic activities also indicates that no link could be found between these functional properties and groups obtained by hierarchical clustering based on the KEGG annotation of the accessory genes.

The literature does not often discuss the gene expression of LAB in relation to its acidifying capabilities. The variations in acidifying potential among different strains, besides the presence/absence of genes, could be due to differential expression of multiple genes related to metabolism. For instance, in a study conducted by Galia et al. (2016), two strains harboring the same allele of the prtS gene, encoding a cell-wall-anchored proteinase linked to fast acidifying capabilities, presented different acidification rates. The authors demonstrated that this difference could be due to the differential expression of several metabolic-related genes (prtS, codY, ilvE, livJ, and relA) during growth in milk, with an over-expression of these genes in the most acidifying strain compared to the other. They also reported that the transcriptional regulator CodY could also play an important role in the acidifying capacity of *Streptococcus thermophilus* through the regulation of nitrogen and carbon metabolism. These conclusions support the results of Lu et al. (2015), who reported that CodY plays an important role in the regulation of cellular processes in *Streptococcus thermophilus* and showed that the global regulator CodY controlled amino acid metabolism and lactose utilization processes. To better understand the phenotype differences of our genetically closed isolates, a thorough study of the expression and regulation of genes involved in carbon and nitrogen metabolism should be conducted.

The lack of a link between the technological capacities and the presence/absence of the genes of the 26 isolates could also simply be due to the low number of studied isolates. Indeed, when it comes to analyzing correlations between phenotypic and genotypic traits of bacteria, the number of samples (or strains) sequenced plays a crucial role in the power to detect correlations. By increasing the number of samples, it would be possible to significantly improve the ability to identify statistically significant correlations.

## 4. Conclusion

Wild strains of *Streptococcus thermophilus* could represent a source of genetic and functional variability from which novel strains or properties might be selected for cheese production. This diversity exists within *Streptococcus thermophilus* isolated at the same location and is not dependent on the season of isolation. A specific technological characterization and safety evaluation of isolates should be carried out to validate the use of this diversity as an autochthonous starter culture.

## Data availability statement

The data presented in this study are deposited in online repositories. Genomes can be found in the EBI repository under the project accession number PRJEB61322. All input and output files of the bioinformatic analysis are available at https://doi.org/10.57745/MSGWXC.

## Author contributions

AG conceived the study, carried out experiments, analyzed the data, performed genome analysis, and wrote the manuscript. ST conceived the study, performed genome analysis, analyzed the data, and participated in the writing of the manuscript. CCa designed the experiments and conceived the project. SH and ED-B supervised the experiments and provided guidance. PG conceived the project and supervised the study. PB and CCh conceived the project, participated in the writing of the manuscript, and supervised the study. All authors contributed to the article and approved the submitted version.

## Footnotes

^1^https://www.inao.gouv.fr/show_texte/4840

^2^https://itol.embl.de/

^3^http://mixomics.org/
