# Supplementary figures and images for: Genetic and technological diversity of Streptococcus thermophilus isolated from the Saint-Nectaire PDO cheese-producing area
Source: Front Microbiol. 2023 Nov 14;14:1245510. doi: 10.3389/fmicb.2023.1245510 (PMC10939066; doi:10.3389/fmicb.2023.1245510)

# PLSDA on the KEGG class genes data

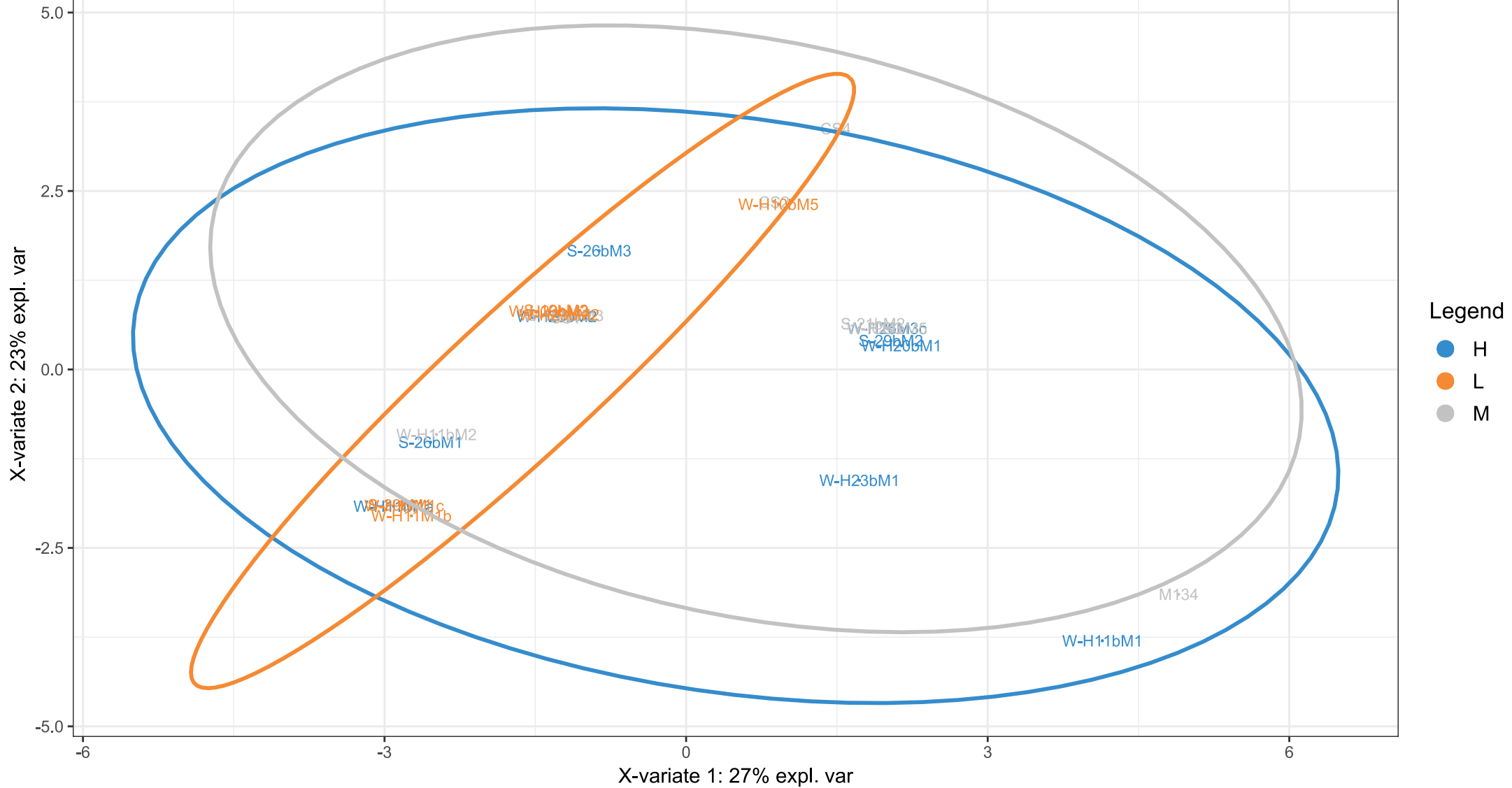

Supplement: Supplementary file 1 [file Data_Sheet_1.PDF]

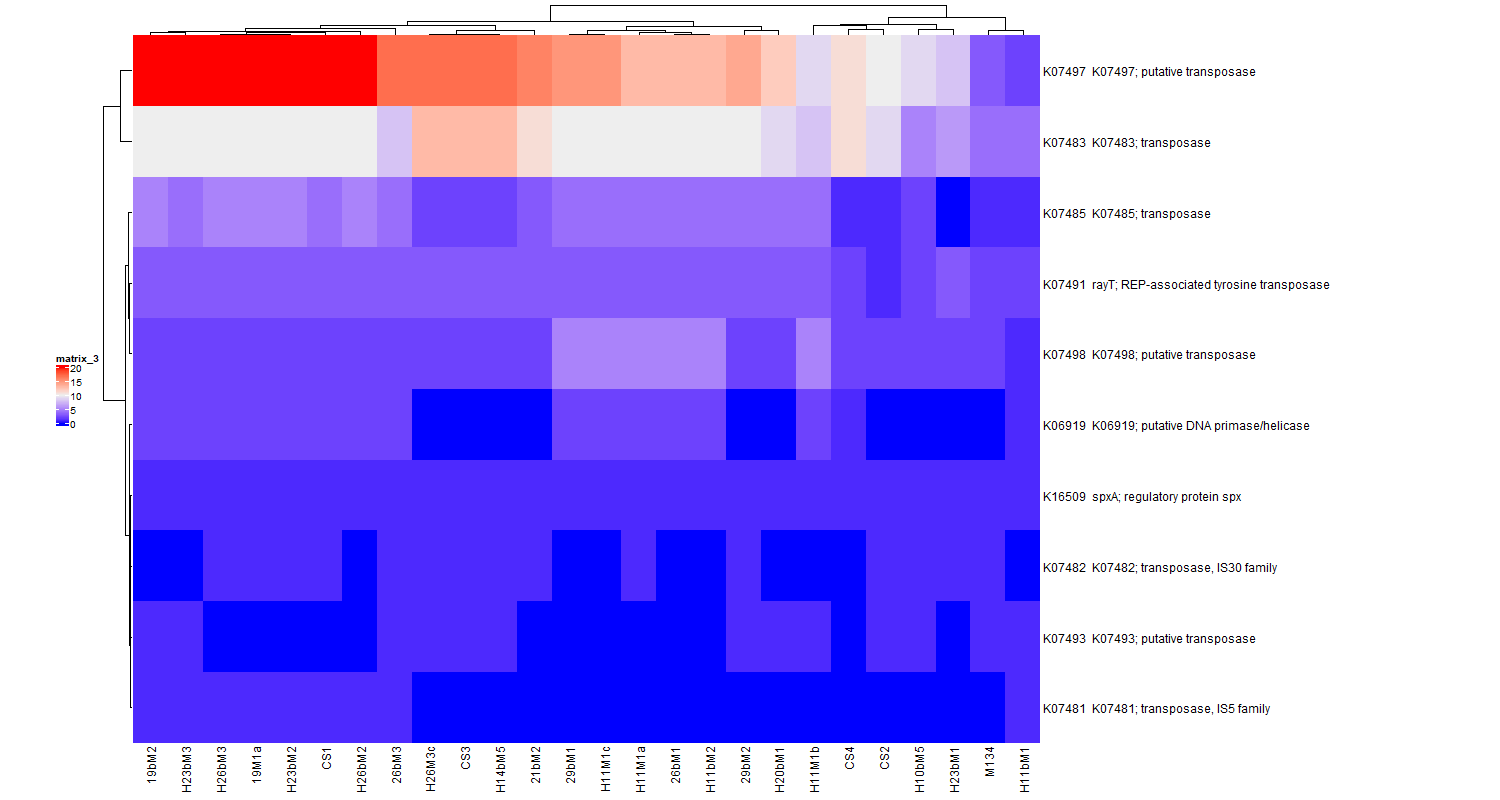

Supplement: Supplementary file 3 [file Image_1.PNG]

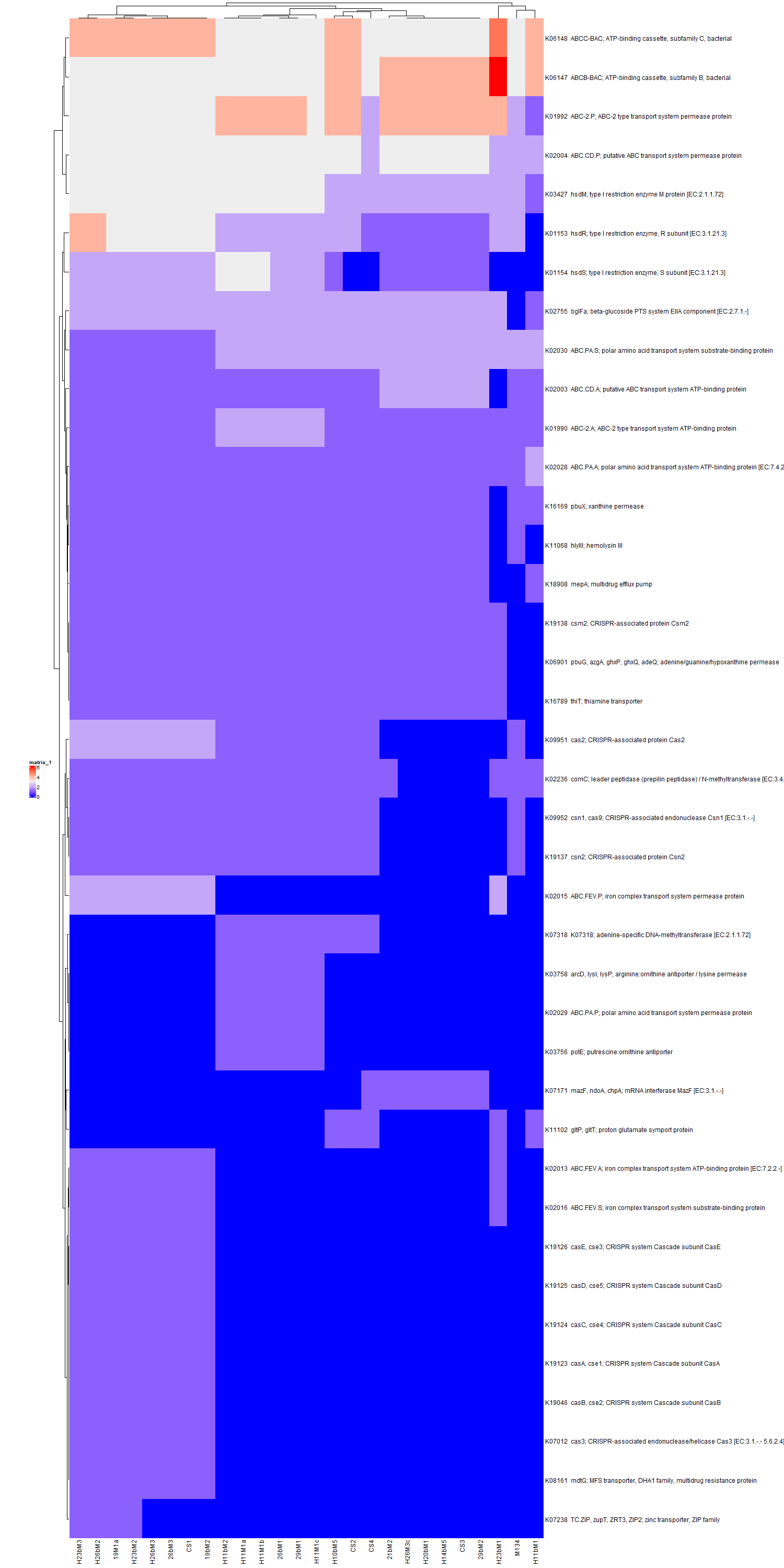

Supplement: Supplementary file 4 [file Image_2.PNG]

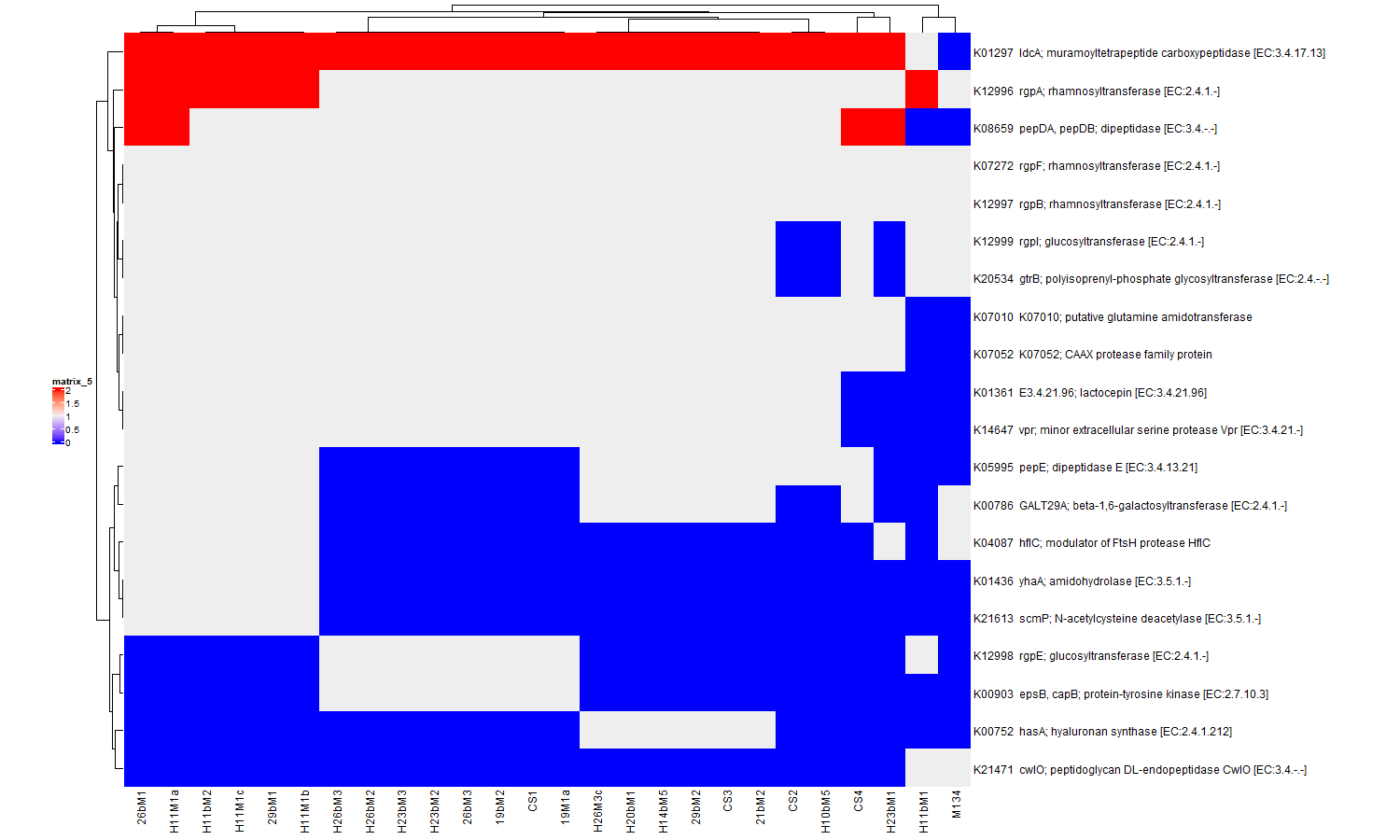

Supplement: Supplementary file 5 [file Image_3.PNG]

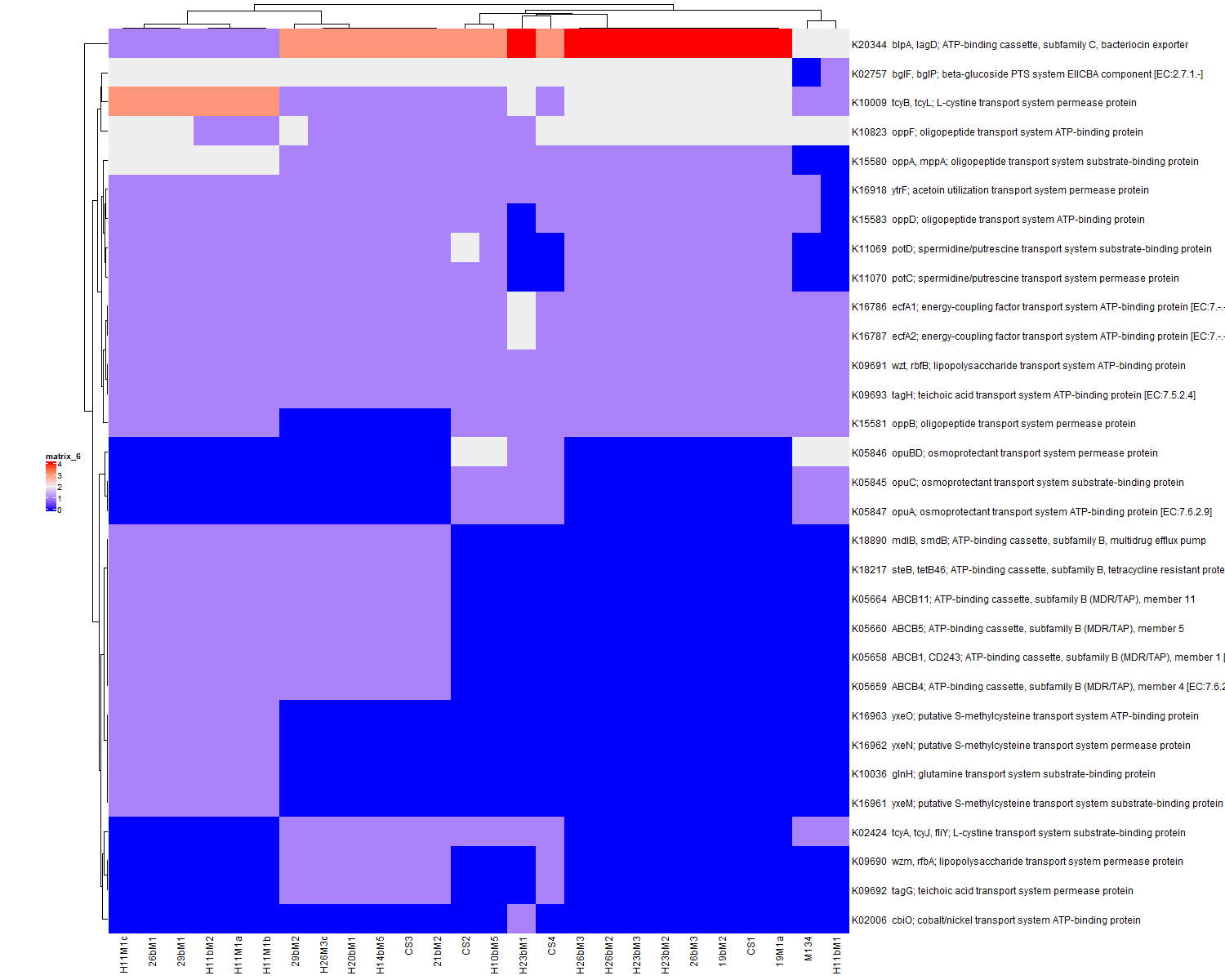

Supplement: Supplementary file 6 [file Image_4.PNG]

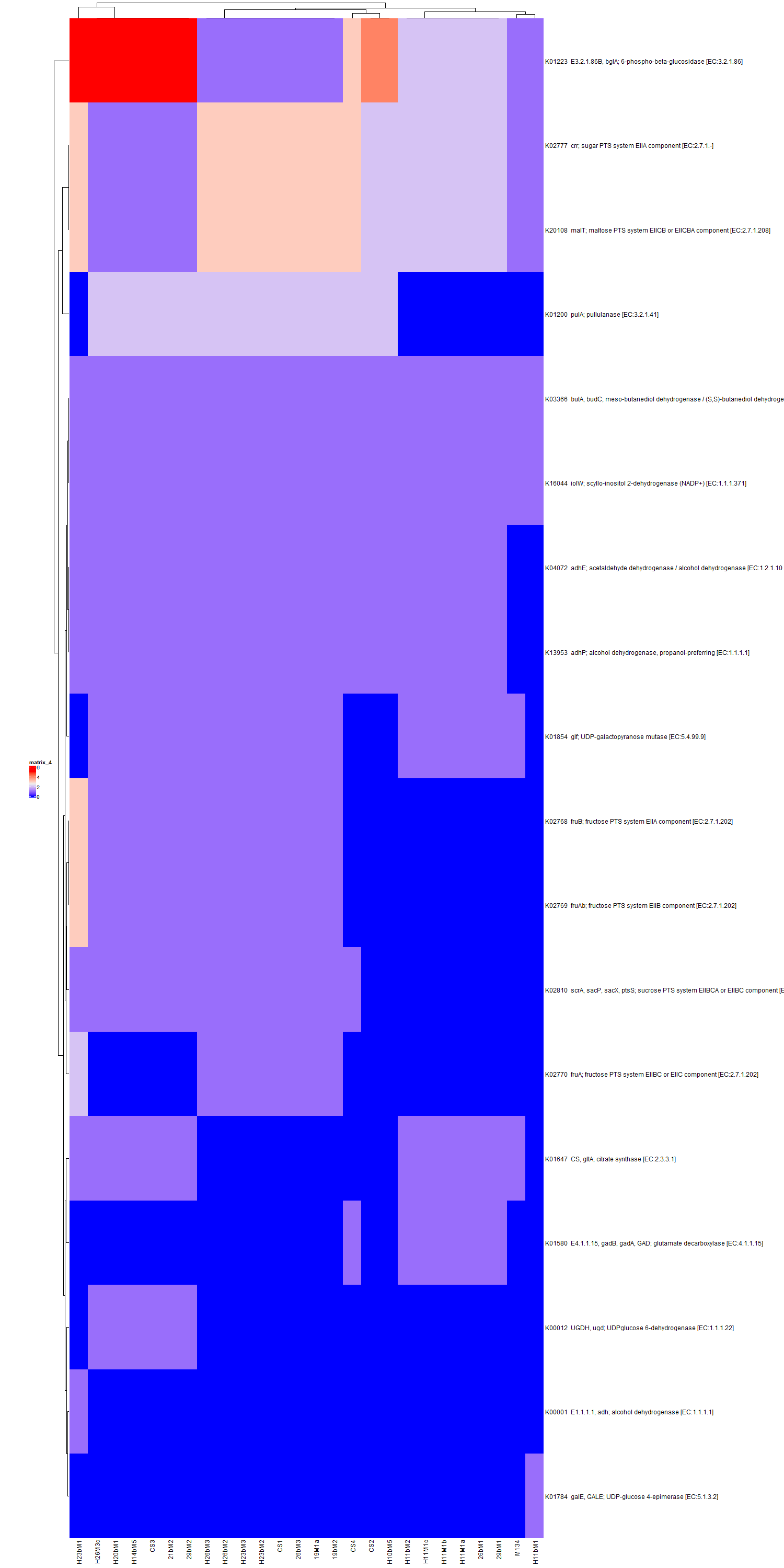

Supplement: Supplementary file 7 [file Image_5.PNG]

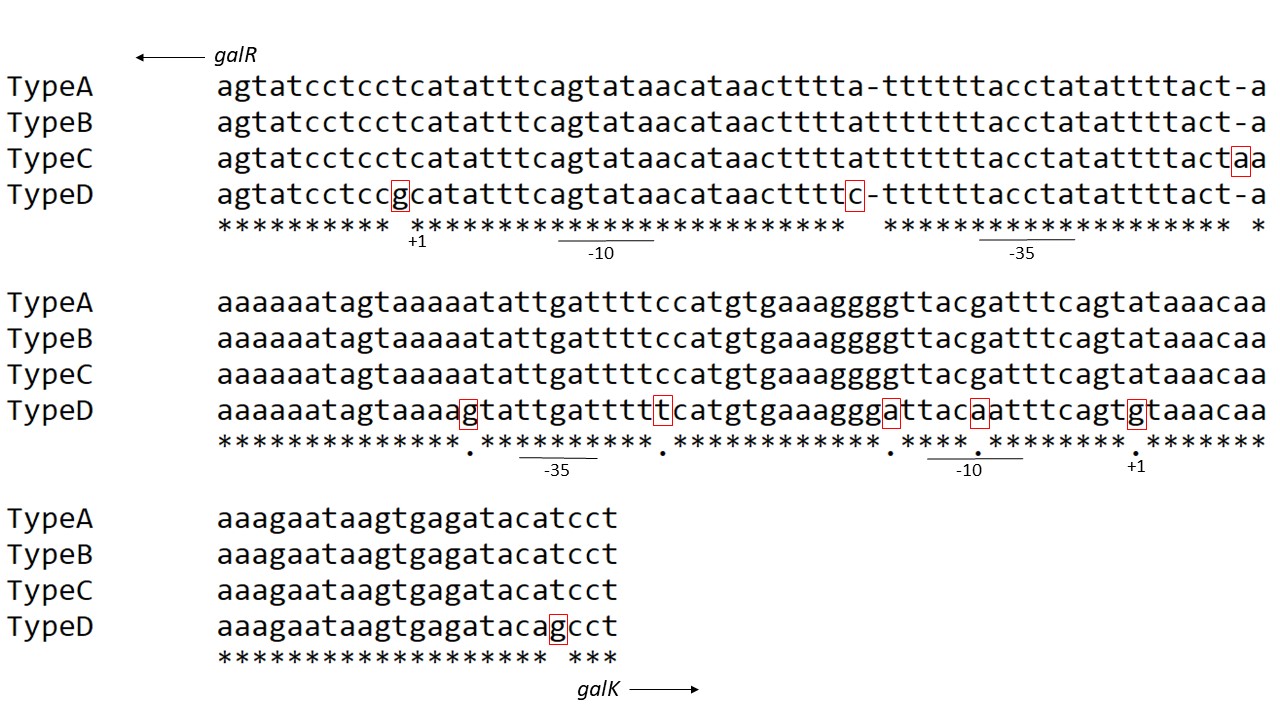

Supplement: Supplementary file 8 [file Image_6.JPEG]
