# Supplementary material for: Genetic and technological diversity of Streptococcus thermophilus isolated from the Saint-Nectaire PDO cheese-producing area
Source: Front Microbiol. 2023 Nov 14;14:1245510. doi: 10.3389/fmicb.2023.1245510 (PMC10939066; doi:10.3389/fmicb.2023.1245510)
Supplement: Supplementary file 2 [file Data_Sheet_2.PDF]

Accessory genes analysis

#Data formatting

#Change the work directory

setwd('/Users/agrizon/Documents/labo/kegg/genes_kegg') dt_genes <- vroom::vroom(file = 'genes.csv', show_col_types = FALSE)

dt_genes_ko <- dt_genes %>% select(KEGG_ko, #query, genome, pan) %>% # We only select columns of interest filter(KEGG_ko != '-') %>% # lines (genes) without kegg annotation are removed mutate(KEGG_ko = strsplit(KEGG_ko, ',')) %>% # KEGG annotation with comma (,) are separated tidyr::unnest(KEGG_ko) # A line for each KO is created

#Only accessory genes are selected and unused columns are removed

dt_genes_accessory <- dt_genes_ko %>% filter(pan == 'accessory') %>% dplyr::select(-c('pan', '#query'))

#KOs are grouped by genomes and their occurence are counted

dt_genes_accessory <- dt_genes_accessory %>% group_by(KEGG_ko, genome) %>% summarise(n= n()) %>% ungroup()

#The table is pivot

dt_genes_accessory <- dt_genes_accessory %>% pivot_wider(names_from = genome, values_from = n) %>% replace(is.na(.), 0)

#'ko:' prefix are removed

dt_genes_accessory$KEGG_k o < -gsub('ko:', '', dt_g enes_a ccessory$KEGG_ko)

#KEGG annotation table is loaded

load('/Users/agrizon/Documents/labo/kegg/ko00001.rda')

#counting table and annotation table are assembled

dt_genes_accessory <- left_join(dt_genes_accessory, kegg_dt, by = c('KEGG_ko' = 'KO'))

#Only the first match is kept

dt_genes_accessory <- distinct(dt_genes_accessory, KEGG_ko, .keep_all = TRUE)

#WE only select "lvlB" of KEGG annotation and sum the genes in the categories

```
dt_genes_accessory_KEGG <- dt_genes_accessory %>% select(-
c(KEGG_ko,lvlC,lvlA,lvlD))%>% group_by_at("lvlB") %>% summarise_each(funs(sum))
dt_genes_accessory_KEGG <- na.omit(dt_genes_accessory_KEGG)
```

#Heatmap at "lvlb" creation

#An heatmap is realized

```
dt_heatmap <- column_to_rownames(dt_genes_accessory_KEGG, var = 'lvlB')
heatmaply(dt_heatmap) dev.off()
```

#PLS-DA ANALYSIS

#Library loading

```
library(vroom) library("readxl") library(mixOmics)
```

```
set.seed(5249)
```

```
dt_genes_accessory_KEGG <- dt_genes_accessory_KEGG %>%
na.omit(dt_genes_accessory_KEGG)
```

#Table representing groups is loaded

```
metadata <- read_excel("~/labo/kegg/metadata.xlsx") X <- t(dt_genes_accessory_KEGG
%>% column_to_rownames('lvlB')) X <- X[, colSums(X) > 0]
```

```
Y <- as.factor(metadata %>% dplyr::select(group) %>% pull)
```

```
dim(X) summary(Y)
```

#PLS-DA

```
res <- splsda(X, Y, ncomp=10)
```

#PlotIndiv

```
plotIndiv(res, title='PLSDA on the KEGG class genes data', ellipse=TRUE, legend = TRUE)
dev.off()
```

#Plot CIM

```
png('plot_cim.png', width=1000, height=1000) cim(res, row.sideColors = color.mixo(Y),
margins = c(20,5), row.cex=0.8) dev.off()
```

#Plot loadings 1 and 2

```
png('plot_loading1.png') plotLoadings(res, comp = 1, contrib= 'max') dev.off()
```

```
png('plot_loading2.png') plotLoadings(res, comp = 2, contrib= 'max') dev.off()
```

#HEATMAPS FOR GENES LEVEL ‘lvlD’

#A filter is added to create a specific heatmap (example to adjust for each ‘lvlB’)

dt_PF_signalling <- dt_genes_accessory %>% filter(lvlB == ‘Protein families: signaling and cellular processes’)

#Only KOs and lowest level (lvlD) are needed for the heatmap

dt_PF_signalling <- dt_PF_signalling %>% distinct(KEGG_ko, lvlD, .keep_all = TRUE)

#An heatmap is created

png(filename = ‘PF_signalling.png’, width = 1500, height = 3000) #Adjust the width and height according to the matrix size hm_PF_signalling <- ComplexHeatmap::Heatmap(dt_PF_signalling %>% ungroup() %>% dplyr::select(where(is.numeric)), row_labels = dt_PF_signalling %>% pull(lvlD), #column_km = 5, # groups number on columns #row_km = 9, # groups numbers on rows) ComplexHeatmap::draw(hm_PF_signalling, padding = unit(c(2, 20, 2, 100), ‘mm’), heatmap_legend_side=“left”) dev.off()
