# Supplementary material for: Genetic and technological diversity of Streptococcus thermophilus isolated from the Saint-Nectaire PDO cheese-producing area
Source: Front Microbiol. 2023 Nov 14;14:1245510. doi: 10.3389/fmicb.2023.1245510 (PMC10939066; doi:10.3389/fmicb.2023.1245510)
Supplement: Supplementary file 9 [file Table_1.DOCX]

Table S1: Municipalities comprised in the Saint-Nectaire cheese PDO area

| Cantal department | | | | |
| --- | --- | --- | --- | --- |
| Allanche | Beaulieu | Champs-sur-Tarentaine-Marchal | Chanterelle | Condat |
| Landeyrat | Lanobre | Lugarde | Marcenat | Marchastel |
| Montboudif | Montgreleix | Pradiers | Saint-Amandin | Saint-Bonnet-de-Condat |
| Saint-Saturnin | Ségur-les-Villas | Trémouille | Vernols |  |
| Puy-de-Dôme department | | | | |
| Anzat-le-Luguet | Ardes | Aurières | Aydat | Bagnols |
| Besse-et-Saint-Anastaise | Chambon-sur-Lac | Chassagne | Chastreix | Compains |
| Courgoul | Cournols | Creste | Cros | Dauzat-sur-Vodable |
| Egliseneuve-d’Entraigues | Espinchal | Grandeyrolles | LaBourboule | La Chapelle-Marcousse |
| La Godivelle | La Tour-d’Auvergne | Labessette | Larodde | Le Vernet-Sainte-Marguerite |
| Mazoires | Montaigut-le-Blanc | Mont-Dore | Murat-le-Quaire | Murol |
| Olloix | Orcival | Picherande | Rentières | Roche-Charles-la-Mayrand |
| Saint-Alyre-ès-Montagne | Saint-Diéry | Saint-Donat | Saint-Genès-Champespe | Saint-Nectaire |
| Saint-Pierre-Colamine | Saint-Victor-la-Rivière |  |  |  |
